# Retinoblastoma: An update on genetic origin, classification, conventional to next-generation treatment strategies

**DOI:** 10.1016/j.heliyon.2024.e32844

**Published:** 2024-06-11

**Authors:** Ashutosh Pareek, Deepanjali Kumar, Aaushi Pareek, Madan Mohan Gupta, Philippe Jeandet, Yashumati Ratan, Vivek Jain, Mohammad Amjad Kamal, Muhammad Saboor, Ghulam Md Ashraf, Anil Chuturgoon

**Affiliations:** a Department of Pharmacy, Banasthali Vidyapith, Banasthali, 304022, Rajasthan, India; b School of Pharmacy, Faculty of Medical Sciences, The University of the West Indies, St. Augustine 3303, Trinidad and Tobago; c Research Unit Induced Resistance and Plant Bioprotection - USC INRAe 1488, University of Reims, PO Box 1039, 51687, Reims, France; d Department of Pharmaceutical Sciences, Mohan Lal Sukhadia University, Udaipur, 313001, India; e Joint Laboratory of Artificial Intelligence for Critical Care Medicine, Department of Critical Care Medicine and Institutes for Systems Genetics, West China School of Nursing, Frontiers Science Centre for Disease-related Molecular Network, West China Hospital, Sichuan University, China; f King Fahd Medical Research Centre, King Abdulaziz University, Jeddah, 21589, Saudi Arabia; g Department of Pharmacy, Faculty of Allied Health Sciences, Daffodil International University, Dhaka, 1207, Bangladesh; h Enzymoics, Novel Global Community Educational Foundation, 7 Peterlee Place, Hebersham, NSW, 2770, Australia; i Department of Medical Laboratory Science, College of Health Sciences, and Research Institute for Medical and Health Sciences, University of Sharjah, Sharjah, 27272, United Arab Emirates; j Discipline of Medical Biochemistry, School of Laboratory Medicine and Medical Sciences, University of KwaZulu-Natal, Durban, 4041, South Africa

**Keywords:** Retinoblastoma, Paediatric, Quadrilateral retinoblastoma, Nanostructured drug delivery systems, Gene therapy

## Abstract

The most prevalent paediatric vision-threatening medical condition, retinoblastoma (RB), has been a global concern for a long time. Several conventional therapies, such as systemic chemotherapy and focal therapy, have been used for curative purposes; however, the search for tumour eradication with the least impact on surrounding tissues is still ongoing. This review focuses on the genetic origin, classification, conventional treatment modalities, and their combination with nano-scale delivery systems for active tumour targeting. In addition, the review also delves into ongoing clinical trials and patents, as well as emerging therapies such as gene therapy and immunotherapy for the treatment of RB. Understanding the role of genetics in the development of RB has refined its treatment strategy according to the genetic type. New approaches such as nanostructured drug delivery systems, galenic preparations, nutlin-3a, histone deacetylase inhibitors, N-MYC inhibitors, pentoxifylline, immunotherapy, gene therapy, etc. discussed in this review, have the potential to circumvent the limitations of conventional therapies and improve treatment outcomes for RB. In summary, this review highlights the importance and need for novel approaches as alternative therapies that would ultimately displace the shortcomings associated with conventional therapies and reduce the enucleation rate, thereby preserving global vision in the affected paediatric population.

## 1 Introduction

The World Health Organization's (WHO) inaugural *world report on vision* issues revealed that over one billion individuals across the globe are currently enduring vision impairment resulting from a multitude of ocular diseases [1]. Often, these diseases remain asymptomatic for prolonged periods of time, which can lead to sufferers being unaware of the condition until it has progressed to an advanced stage, rendering the treatment ineffective.

One such disease is retinoblastoma (RB), a rare yet malignant paediatric intra-ocular cancer [2]. The disease presents as an aggressive tumour in the retina, arising from the precursors of the cones, and is primarily found in children under five years old [[3], [4], [5]]. It affects approximately 1 in 16,000–18,000 new-borns globally [6]. The retinoblastoma gene (*RB1)* on chromosome 13 inhibits rapid and uncontrolled cell growth. Biallelic mutations in the *RB1* gene lead to RB development [7]. Unfortunately, the burden of RB is mostly concentrated (>80 %) in low- and middle-income countries (LMICs), where the prognosis is comparatively poorer than in high-income countries (HICs). This is attributed to inadequate awareness about the disease and a lack of trained ocular oncologists, ultimately leading to delayed diagnosis [8]. Recent studies have shown that RB incidence is highest in Asian countries (53 %), followed by Africa (29 %), with minimal occurrence in North America (3 %) [9].

Early manifestations of RB include several identifiable signs, the first being an abnormal white retinal reflex termed leukocoria or cat's eye. Leukocoria transpires when the presence of a tumour causes light entering the eye to reflect in the pupil. Another observable sign is the misalignment of the eyes, known as strabismus, which may develop following the occurrence of leukocoria [10,11].

RB treatment strategies are constantly evolving, providing more promising outcomes for those affected worldwide. This review presents the epidemiology of RB, current categorization methods, and traditional treatment strategies, along with an in-depth discussion of their shortcomings and potential improvements offered by innovative drug delivery systems and treatment strategies. Furthermore, the review highlights new therapeutic agents that may usher in a new era in RB treatment and provides an overview of the completed and ongoing clinical trials.

## 2 Method used

Only articles published between 1994 and 2023 were considered for this study, with a specific focus on RB aetiology, conventional and recent treatment strategies, and novel drug delivery systems. Articles that were available in the English language with the search keywords "management of RB," "tumours," "nanoparticles," "chemotherapy," "enucleation," "epidemiology," "recent management strategies," "paediatric cancer" were obtained from various databases, namely PubMed, Medline, National Organization of Rare Diseases (NORD), ScienceDirect, Cancer, Scopus, and WHO.

## 3 Classification of RB

### 3.1 Based on the affected site

a. Unilateral RB; is characterized by the occurrence of a single tumour in one eye and accounts for approximately 60–70 % of all reported cases.
b. Bilateral RB; affects both eyes and is characterized by the presence of a multifocal tumour. Bilateral RB constitutes only 5 % of the total cases reported to date [12].
c. Trilateral RB; a rare and lethal form of RB, is characterized by the development of a tumour intracranially in the pineal gland, located at the base of the brain. The occurrence of this tumour is closely linked to bilateral RB and because of its location it is known as pineoblastoma. It is more likely to develop when a child displays hereditary RB [13].
d. Quadrilateral RB; is an exceedingly rare and fatal form of the disease. It is characterized by the spread of the tumour from the pineal gland to the entire brain, along with bilateral RB [14].

### 3.2 Based on the extent of the spread lesion

a. Intraocular, if the tumour is confined to the retina.
b. Extraocular, if the tumour spreads to the tissues surrounding the eye; the survival rate usually decreases with tumour spread [15].

### 3.3 Classification schemes

The classification schemes for RB have changed with advances in therapeutic options.

#### 3.3.1 Reese and Ellsworth classification

In the 1960s, Reese and Ellsworth (R-E) developed a rudimentary classification system to manage RB, when external beam radiation therapy (EBRT) was the primary treatment option. This system, comprised of five groups (I – V), was used to predict clinical outcomes following EBRT. However, with the advent of chemotherapy, this classification system was deemed insufficient, particularly in describing the course of vitreous seeding [16].

#### 3.3.2 International Intraocular RB classification

Tumour classification was redefined in 2005 by introducing the International Intraocular RB Classification (IIRC). It divides RB into five groups, Group I - V, based on the extent of tumour spread and size and other tumour-associated features [17]. The IIRC provides a more comprehensive and accurate classification system, enabling a better understanding of the disease and improving treatment strategies.

#### 3.3.3 International RB Staging System

RB was classified from 0 to IV according to the International RB Staging System (IRSS) devised in 2006, where stage 0 indicates the intraocular spread of the tumour while stage IV describes the metastatic phase of RB. Stage IV is likely to have a delayed prognosis [18].

#### 3.3.4 Intraocular Classification of RB

The IIRC was introduced in 2005 to classify RB based on the spread and size of the tumour and its associated features. However, the IIRC was inadequate in explaining the characteristics of groups D and E, which led to the development of the Intraocular Classification of RB (ICRB) in 2011 [16]. The ICRB provides improved information on the characteristics of tumours related to groups A to E. Table 1 describes the stages of IIRC and ICRB along with the characteristics and therapies for the progressive groups [18]. IIRC and ICRB systems emphasize the use of systemic chemotherapies for progressive stages of RB, which are widely accepted by physicians and researchers as valuable tools for managing this disease [19].

**Table 1:** The international intraocular retinoblastoma (RB) classification.

Type of Tumour	Stage of Tumour	Risk Extent	Tumour Characteristics as per IIRC	Tumour Characteristics as per ICRB	Therapy Employed	Diagnostic representation of tumour
Group A (Small tumours)	Slight advanced unilateral	Slight risk	• Tumour size ≤ 3 mm thick. • The tumour is restricted to the retina and away from the optic disc and foveola. • Absence of vitreous and sub-retinal seeds.	• Tumour size ≤ 3 mm thick.	Brachytherapy, Thermotherapy/Cryotherapy, Laser photocoagulation	
	Slight advanced bilateral				Intravenous chemotherapy	
Group B (Large tumours)	Sight advanced unilateral	Low risk	• Size is larger than 3 mm. • Retinal tumours are found close to the optic disc and foveola.	• Tumour thickness >3 mm. • ≤ 3 mm towards the foveola at the macular location. • ≤ 1.5 mm towards the disc at the juxtapupillary location. • Subretinal fluid additionally at ≤ 3 mm from the margin.	Brachytherapy, Thermotherapy/Cryotherapy, Laser Photocoagulation, Intravenous/Intraarterial chemo reduction	
	Slight advanced bilateral				Brachytherapy, Thermotherapy/Cryotherapy, Laser Photocoagulation, Intravenous chemotherapy	
Group C (Contiguous seeds)	Slight advanced unilateral	Moderate Risk	• Presence of slight seeding in the vitreous humour and subretinal fluid of ≤3 mm • Uncertain size specification for the tumours.	• Presence of subretinal seeding ≤ 3 mm around the tumour. • At ≤ 3 mm from tumour presence of vitreous seeds.	Intra-arterial/Intravitreal chemotherapy, Focal therapy	
	Slight advanced bilateral				Focal therapy, Intravenous chemotherapy	
Group D (Diffuse seeds)	Advanced unilateral	High risk	• More diffuse vitreous and subretinal seeding. • Seeds are more extensive than the Group C ones. • Tumours could be either exophytic or endophytic.	Both subretinal and vitreous seeds present > 3 mm from the tumour.	Intraarterial/Intravitreal chemotherapy and Enucleation	
	Advanced bilateral				Periocular Chemotherapy, intravenous chemotherapy, and enucleation	
Group E (Extensive tumour)	Advanced unilateral	Very high risk	• Destruction of the retina due to extensive tumour growth till the lens. • Growth of the tumour reaches the anterior portion of the eye along with neovascular glaucoma. All these factors result in massive ocular haemorrhage.	• Extensive tumours occupy more than 50 % of the ocular globe, resulting in neovascular glaucoma. • Ocular media turns opaque due to haemorrhage in both the vitreous and anterior chamber along with the subretinal space. • Tumour invades the optic nerve choroid at >2 mm, sclera, orbit and the orbit chamber.	Periocular therapy and External Beam Radiation Therapy	
	Advanced bilateral				Enucleation, Intravenous therapy, Intra-arterial chemotherapy	

#### 3.3.5 TNMH classification

The American Joint Committee on Cancer (AJCC) introduced a new RB categorization system in 2018, known as the 8th edition of the TNMH scheme [20]. This scheme classifies tumours according to their stage, lymph node involvement, metastasis, and heritability [21]. It provides a clear pathologic progression from group 0 to group 4 and describes metastases in the extraocular regions. Because it includes all the clinical characteristics of the tumour and indicates its location, whether intraretinal, intraocular, or extraocular [22], it has been termed the cTNMH scheme, where c stands for clinical. In addition, it also considers the hereditary status of the tumour and has the potential to become a reference for future research on RB [23].

#### 3.3.6 RSU classification

A novel classification scheme, known as the Retinoblastoma Seeding and Uveal (RSU) classification, was developed to enhance the prognostic accuracy for RB recurrence. The RSU classification system categorizes RB recurrences based on three criteria: retinal involvement, extraretinal seeding, and uveal involvement. Considering these factors, the RSU classification system can provide insight into potential relapse outcomes that may manifest within 2–3 months of treatment cessation. It is important to note that RSU classification does not differentiate between the recurrence of the previously inactive tumour and the development of new tumours. In severe cases, secondary enucleation may be recommended as the preferred course of action [24]. Nonetheless, the RSU classification system represents a significant step forward in predicting the recurrence of RB and facilitating more informed treatment decision-making.

## 4 Genetic origin

**Fig. 1:**
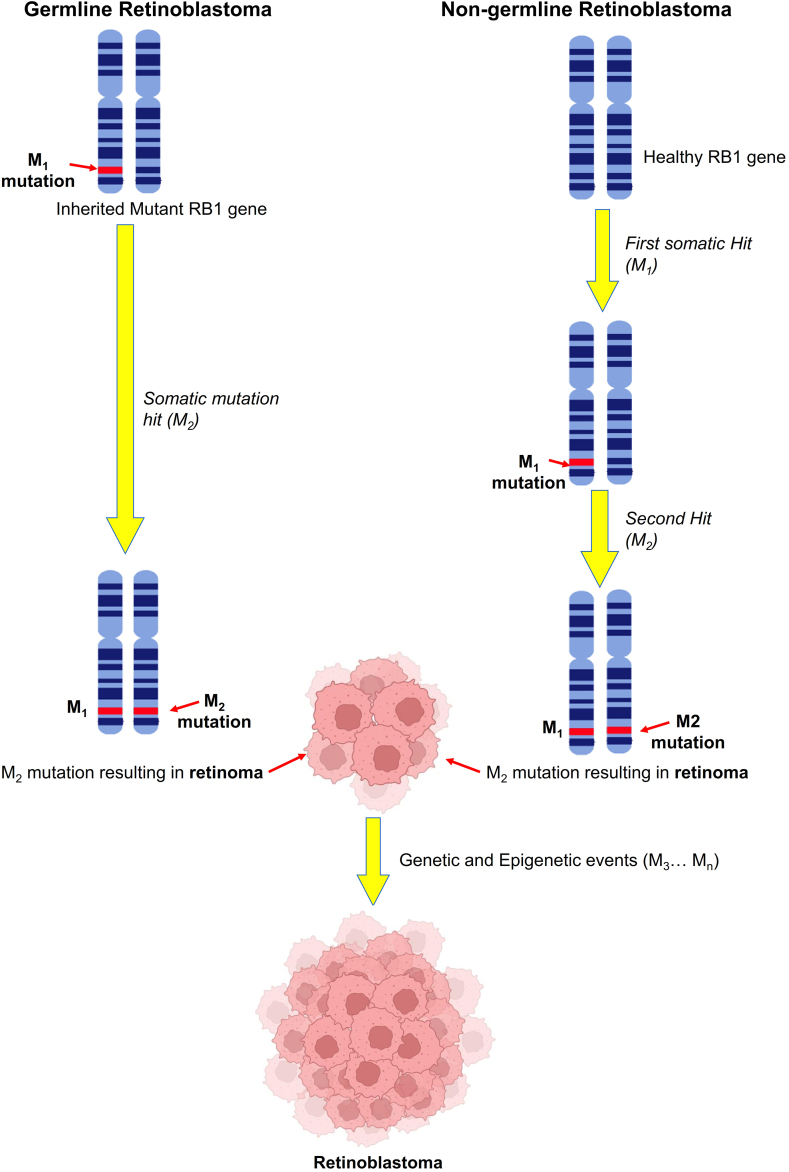
Genetic Origin (In germline retinoblastoma, also known as hereditary retinoblastoma, the mutation in the first allele of the *RB1* gene is inherited, M1. Upon somatic mutation hit, the second allele of the *RB1* gene undergoes mutation, M2. The second mutation results in the formation of retinoma. In non-germline RB, also known as non-heritable one, the first mutation in the allele of the *RB1* gene resulted in an M1 mutation. M2 mutation occurred upon somatic hit, which ultimately formed retinoma. The genetic and epigenetic events transformed retinoma into retinoblastoma).

Gene mutations play a prominent role in the aetiology and onset of RB. The *RB1* gene, located on chromosome 13q14 [25], serves as a key regulator of cell growth and controls cell division by binding to the E2F transcription factor 1 [26]. Biallelic inactivation and subsequent mutations in the *RB1* gene disrupt this intricate process and the proteins associated with its expression. The resulting negative regulation of cell proliferation and differentiation by the RB protein is lost, leading to uncontrolled and aggressive cell growth that manifests as retinoma [27]. The complex interplay of genetic and epigenetic alterations further contributes to the development and progression of the tumour (Fig. 1) [28].

The pathogenesis of RB is multifactorial and involves numerous pathways. Genetic mutations, such as premature termination codons or splicing disruptions, can introduce out-of-frame skipping of exons, leading to the development of this chronic disease. In the follow-up studies on the DNA of affected individuals, approximately 2500 nucleotide variants suspected to cause the genetic mutations have been identified. In addition to the *RB1* gene, cancer-predisposing genes *NTHL1* and *MSH3* have heterozygous mutations in their base excision repair and are responsible for autosomal recessive cancer predisposition syndromes, thereby acting as oncogenic drivers [29]. On the other hand, the downstream effects of mutated *RPTOR* and *FAT-1* are yet to be discovered [30]. The combination of nucleotide variants, splice site variants, and abnormal DNA reorganization are *RB1* gene mutations. Depending on the type of gene alterations, RB can be classified as.

a. hereditary or familial hereditary RB
b. sporadic hereditary RB
c. mosaic RB
d. nonhereditary RB

*a. Hereditary or familial hereditary RB* – Familial RB, a type of hereditary RB, is characterized by the inheritance of an autosomal dominant trait that augments the possibility of developing this tumour. RB screening within the family context can be commenced before or during pregnancy and is typically associated with the pre-symptomatic diagnosis [31].There are two types of penetrance in hereditary RB: complete and incomplete. Complete penetrance indicates that at least one RB centre arises in every heterozygous family member for an oncogenic *RB1* allele. On the other hand, even if some family members are heterozygous carriers of an oncogenic *RB1* allele, hereditary RB has incomplete penetrance, resulting in some family members being tumour-free [32].

*b. Isolated hereditary RB* – Isolated hereditary RB can occur in children of apparently healthy parents. This can happen when a *de novo* prezygotic mutation arises in the germ cells of one of the healthy parents, leading to the development of RB in the offspring. The characteristics of the original mutation and expression of the number of primary tumour foci determine whether it is an isolated bilateral RB or RB with genomic deletion of chromosome 13q14 [33].

*c. Mosaic RB* – Mosaic RB refers to a distinct form of RB that typically presents as unilateral disease in the initial affected family member and subsequently progresses to bilateral disease in later generations. Some affected individuals are mosaics of the mutant *RB1* allele. In some instances, individuals with mosaic RB exhibit a mutant sector, which arises due to a mutation that originates during early intrauterine development in the affected individual. There are expected to be fewer tumour foci in these patients, possibly because the mutant sector makes the development of RB less likely in an individual with somatic mosaicism [34].

*d. Nonheritable RB* – In approximately 50 % of newly diagnosed RB cases, only one eye is affected, and there is no prior family history of the disease. RB DNA genetic testing can reveal alterations in both *RB1* alleles in nearly 90 % of these patients [35]. Alterations in the first *RB1* allele (M1) occur in almost every cell in the body. With mutations in the second allele (M2), these mutations render the tumour benign and are referred to as hereditary RB [36]. This type of RB is readily passed down to offspring and has the tumour potential to manifest as bilateral and multifocal tumours. However, in some cases, the tumours may be unilateral and unifocal. Children with this form of RB are at high risk of developing other cancers later in life [35].

Nonheritable RB is characterized by the presence of two mutated tumour suppressor gene alleles. Still, unlike hereditary RB, the mutations M1 and M2 are limited to the retinal cell only (as depicted in Fig. 1). It is worth noting that there are rare instances where non-germline issues arise due to the amplification of the *MYCN* oncogene while the *RB1* genes remain unaltered [37].

## 5 Deregulated signalling pathways of RB

Unraveling the genetic basis of retinoblastoma has revealed a range of causative mutations, clarifying their role in disease onset and their potential connection to diverse clinical presentations and disease severity. The etiologic condition of RB resembles a symphony of dissonance where the deregulation of multiple signalling pathways collectively contributes to the uncontrolled aggressive growth of the tumour [38].

**a. RB tumour suppressor pathway-** The RB tumour suppressor pathway occupies a pivotal position as the driving force for RB pathogenesis by acting as a critical conductor of the cell cycle and its aberrant regulation. As discussed earlier, the *RB1* gene serves as the architect, encoding the retinoblastoma protein (pRB), a crucial tumour suppressor that governs the orderly progression of the cell cycle; thus, this pathway is regarded as the master regulator in RB pathogenesis. pRB acts as a molecular custodian which binds to transcription factors E2F proteins, arresting the cell cycle. Aberrations within the *RB1* gene, encompassing both deleterious mutations and deletions, culminate in the abrogation of functional pRB protein, which initiates an unrestrained cell proliferation, resulting in tumour formation and ultimately leading to RB [39]. Furthermore, the delicate equilibrium the RB pathway maintains is contingent upon the proper functioning of other genes like *CDK4, CCND1, MYCN* [39] and *CDKN2A* [40]. Dysregulation of these genes can not only disrupt the pathway but also potentially exacerbate retinoblastoma development through a multifaceted interplay.

**b. p38-MAPK Pathway –** The Mitogen-Activated Protein Kinase (MAPK) pathways act as a highly regulated signalling cascade that is adept at transmitting, amplifying and integrating a range of stimuli [41]. As a result, it elicits cellular differentiation, proliferation, retinal development, maintaining cellular homeostasis, inflammatory cascades and even programmed cell death [42]. As per the latest research, TRIM59 (Tripartite motif-containing protein 59) has been the driving force in the progression of RB [43]. The upregulation of TRIM59 promoted aberrant cell proliferation and differentiation through the p38-MAPK pathway. It also influenced cell-cycle progression by facilitating thee transition from the G1 to S cell-cycle phase. Attacking the MAPK pathway suppresses the apoptotic function of this pathway and fuels the aggressive, uncontrolled growth of retinal cells. The other proteins mutated by the protein attack on the MAPK pathway are yet to be discovered clinically [43].

**c. Notch Signalling Pathway –** This pathway is pivotal for a myriad of cell fate decisions during embryonic development, involving proliferation, differentiation, and intracellular communication, especially in the retinal cell types [44,45]. This pathway is an interplay between Notch receptors (Notch 1–4) and Notch ligands (Jagged 1&2, Delta) [46]. It has been identified that, during retinal development, Notch receptors suppress the differentiation of photoreceptors and maintain the progenitor state of the forming retinal cell types. Notch receptors are able to function normally owing to the successful binding of the Notch ligands to its cognate receptors, which leads to a series of consecutive proteolytic cleavages of the receptor. The cleavages, in turn, trigger the release of the Notch intracellular domain (NICD), transmitted by the γ-secretase complex. NICD combines with the transcriptional factor CBF/Su(H)/LAG and MAML (Mastermind-Like) as it is translocated in the nucleus. Upon formation of the complex, Notch target genes *Hes* (*Hairy and enhancer of split*) and *Hey* (*Hes-related repressor protein)* are activated, which binds to specific DNA sequences, initiating the transcription of genes for normal cellular functions [45]. Mutations derail the normal functioning of the Notch pathway. Mutations in the Notch receptors, Notch ligands, or the Notch targeting genes can lead to abnormal cellular processes [44]. Downregulation of the ligands (Jagged) due to mutations may lead to disrupted signalling from the Notch receptors, causing improper cell differentiation and forming a mass of cells. Conversely, upregulation of those ligands will upsurge the pathway activation, fostering unrestrained cell proliferation [44]. Further clinical research to analyse the range of mutations that contribute to the dysfunctioning of this pathway in RB will be promising for developing targeted therapeutic delivery strategies [46].

**d. pI3K/AKT pathway** - The phosphoinositide 3-kinase (PI3K)/v-akt murine thymoma (AKT) pathway has a regulatory influence on multiple vital cellular processes, viz., cell growth, cell proliferation, cell metabolism and apoptosis [47]. Several tumour-promoting factors are involved in the abnormal activation of this pathway, involving mutations and loss of alleles [48]. A tumour suppressor gene *PTEN* (Phosphatase and tensin homolog) [48], when it undergoes methylation, phosphorylates the p13 kinases, resulting in abnormal activation and phosphorylation of AKT [49]. PIK3CA (phosphatidylinositol 3-kinase catalytic subunit) encodes for the p110 alpha catalytic subunit of PI3K [47]. PIK3 is one of the vital enzyme units in the regulation of cellular functions [49]. Upon its upregulation, the p110 alpha protein is excessively amplified, which acts as the mediator for antiapoptotic signalling and is the driver for hyperproliferation and apoptosis resistance [50]. Additionally, the activation of PIK3 by Ras, in turn, activates the pI3K/AKT pathway, thereby contributing towards the aberrant cellular functions, ultimately leading to RB [47].

**e. p53 pathway**- This pathway is regarded as the ‘genome’ guardian’ as the p53 protein exerts multiple cellular responses in order to safeguard the genomic integrity [51]. p53 is usually activated upon cell stress activities, like DNA damage, nutrient deficiency and uncontrolled cell cycle processes. After being activated, it is preserved by cell cycle arrest, apoptosis, DNA repair, autophagy, and other molecular processes that inhibit tumorigenesis [51,52]. There are several mechanisms through which this pathway is inactivated. Most often, the upregulation of the *MDM2* gene interferes with the p53 pathway [53] and cuts down the required amounts of p53 protein by proteolysis of p53 protein, thereby causing cells to proliferate aggressively [54]. It is considered to be one of the key oncogenic regulators and has been proven to promote retinal cancer [54]. Another gene that undergoes mutation to disrupt this pathway is *TP53*. Mutations or deletion of this gene in the developing retina inactivates the protein p53, thus making the latter lose its ability to repair cell cycle arrest and DNA [55].

In the realm of RB treatment, where these dysregulated pathways bring a paradigm shift in the treatment options, a plethora of conventional therapeutic strategies hinge upon varied factors, including the size and severity of the RB tumour.

## 6 Conventional management strategies for RB

There are currently myriad treatment options available for RB aimed at preserving vision. The choice of treatment depends on the type and stage of the cancer. Early tumour detection is critical for effective treatment as it prevents the tumour from spreading to surrounding tissues.

**Fig. 2:**
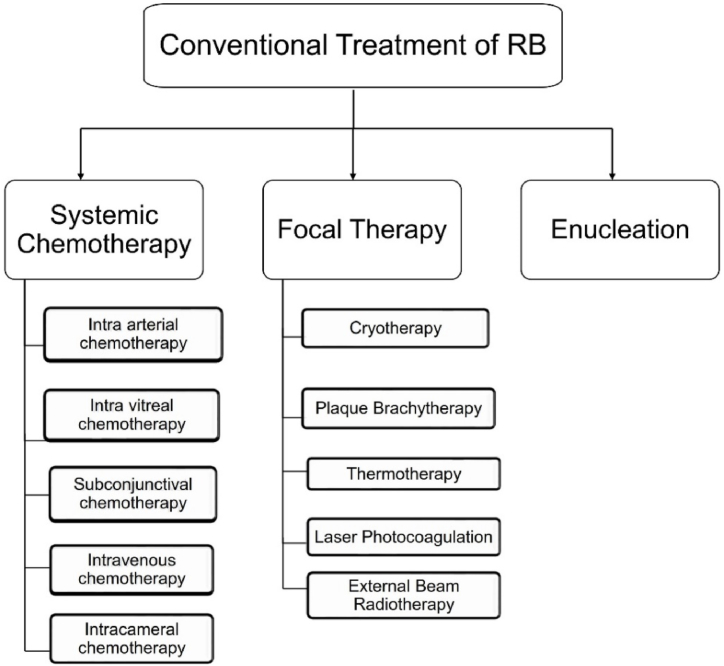
Chemotherapeutic strategies utilized for retinoblastoma.

Currently, the most common treatment options include systemic chemotherapy [56], enucleation [57] and focal therapy [58], as shown in Fig. 2.

### 6.1 Systemic chemotherapy

Systemic chemotherapy is often the primary treatment for RB. The vincristine, etoposide, and carboplatin regimen is usually employed in chemotherapy, either individually or in combination [59]. Other drugs, such as melphalan and cyclophosphamide, can also be used in combination [60].

However, delivering a drug to the posterior segment of the eye is challenging due to the structural and physiological complexity and characteristics of the eye. Ocular barriers, such as the blood-retinal barrier, can significantly affect the drug's pharmacokinetics, potentially reducing the drug's effectiveness in reaching the intended site of action.

#### 6.1.1 Intra-arterial chemotherapy (IAC)

In the intra-arterial chemotherapy approach, chemotherapeutic agents are administered directly to the eye via a transfemoral catheter into the main artery, *i.e.*, the ophthalmic artery [61]. This method is considered the most suitable treatment option for unilateral and non-hereditary RB [62]. The drugs used in this approach may include a combination of drugs or a single drug, such as melphalan, carboplatin, topotecan, and vincristine [63]. Transarterial chemotherapy administration is done using a pulsatile fashion, where the drugs are delivered in short pulses over a period of 30 min [64]. The drug administration is targeted directly to the tumour; therefore, the required dose is comparatively lower than that required for systemic chemotherapy [65]. This would certainly help to minimize the adverse effects of chemotherapeutic agents.

*Challenges:* IAC also has some limitations that need to be considered. Firstly, experienced surgeons are required to perform IAC successfully, which may limit its availability in some areas. Moreover, due to its high cost, IAC may not be the preferred treatment option compared to enucleation, particularly in many developing countries with limited resources. Furthermore, medication complications can negatively affect general and/or eye health, with general anaesthesia, such as bronchospasm, being the most common adverse effect [66].

#### 6.1.2 Intravitreal chemotherapy (IVitC)

This therapeutic modality is most appropriate in cases where the accumulating tumour cells, known as vitreous seeds, have not shown significant improvement after conventional therapies or in cases where the tumour has recurred [67]. The therapy involves delivering the drug through *pars plana* into the area behind the lens to prevent the tumour from spreading. It is crucial to freeze the needle while withdrawing it to avoid tumour seeding [68]. The drugs used in this therapy include melphalan and topotecan, administered either individually or in combination. It can also be applied in conjunction with IAC or separately [69].

*Challenges Involved -* Extraocular tumour expansion and metastatic risk are the key concerns associated with intravitreal chemotherapy. After intravitreal administration, the internal limiting membrane (ILM) barrier effect can limit the drug's effectiveness due to its diminished delivery to the retina. This thin membrane barricades the vitreous humour and retina [70]. The ILM obstructs the pathway of the positively charged drug molecules, while neutral and negatively charged molecules reach the retina smoothly. This is because the ILM is negatively charged and favours the entry of similarly charged molecules [71]. Drug molecules with pore sizes larger than the ILM pore size tend to impede permeation [72]. Therefore, optimising the drug's physicochemical properties and selecting the appropriate administration route is critical to ensure effective treatment.

#### 6.1.3 Subconjunctival chemotherapy

In this approach, the drug is delivered directly under the conjunctival membrane to bypass the epithelium. This therapy is often used in conjunction with a reduction in systemic chemotherapy to achieve a high concentration of the drug intraocularly in cases where the RB has reached an advanced stage, with extensive tumour spread in the vitreous and seeding on the retina [73]. In a study by Abramson et al. [74], carboplatin was administered subconjunctivally at a 10–20 mg dose thrice monthly after subjects underwent two chemo-reduction cycles. The researchers observed a 10-fold increase in the vitreous concentrations of carboplatin [74].

*Challenges*: However, the major drawback of subconjunctival chemotherapy is its inability to suspend the seeding of subretinal tumours effectively [75].

#### 6.1.4 Intravenous chemotherapy (IVC)

Intravenous chemotherapy is the most widely used therapy for RB and is typically used in conjunction with focal therapies, which have been shown to improve drug availability and potentially eliminate the need for radiotherapy and enucleation. IVC is primarily used to treat bilateral RB. The likelihood of metastasis also decreases with the use of IVC. However, the blood-retinal barrier (BRB) poses a significant challenge to the therapeutic efficacy of drug molecules reaching the target site. Furthermore, intravenous chemotherapy can cause systemic side effects such as nephrotoxicity, bone marrow suppression, and other adverse effects [76]. To reduce tumour volume effectively, the treatment regimen typically involves administering a combination of carboplatin, vincristine, and etoposide [77].

*Challenges involved* - The BRB is a significant obstacle to drug delivery across the IVC. BRB comprises two types of cells: retinal capillary endothelial cells and retinal pigment epithelial (RPE) cells, which form the inner and outer BRB, respectively. The BRB acts as a selective barrier, restricting the transport of drugs between the neural retina and the systemic circulation. Due to the presence of tight junctions, it is inherently restrictive and responsible for the transport of various ions, proteins, and aqueous outflow through the retina [78]. The drug molecule permeability through the BRB depends on various physicochemical factors, including the concentration of the administered drug, the volume of distribution, its binding to plasma protein, and targeting efficiency. The size of drug particles also significantly influences their transport through the barrier. Smaller molecules can cross the tight junctions of the RPE without hindrance, while the permeability of larger molecules is hindered. In such cases, the role of transporter molecules, such as folate and amino acids, becomes crucial [79].

#### 6.1.5 Intracameral chemotherapy

In the case of unilateral group ERB, aqueous seeding remained incurable with other delivery methods, leading to the consideration of intracameral drug administration, explicitly targeting the anterior and posterior chambers to achieve the desired drug concentration. In this technique, first and foremost, the aqueous volume is entirely aspirated from both chambers and then melphalan is injected through the cornea into the chamber to control aqueous seeding. During the melphalan injection process, ciliary secretion is restrained to prevent dilution of the administered drug. Further, a tumour-free meridian is selected by ultrasonic biomicroscopy (UBM) to perforate the iris root, targeting both anterior and posterior chambers. *Trans*-iridial injection to the vitreous chamber prevents cross-contamination between the anterior and the posterior chambers. This therapy has been found to preserve vision much better than previously described chemotherapies [80].

### 6.2 Focal therapy

This therapy encompasses a range of treatments that selectively ablate the tumour while minimizing damage to the surrounding tissues [81]. In instances where the tumours are relatively small, confined to the retina, and have not spread to the eye, focal therapy may be used. However, when tumours are large and have extensively spread throughout the retina, causing retinal detachment, it is prudent to combine focal therapies with either IVC or IAC [82].

#### 6.2.1 Cryotherapy

Several studies suggest that cryotherapy is highly effective for tumours that are confined to the retina. A single cryotherapy application is sufficient for tumours with a diameter of 1.5 mm, while one or more cycles are required for tumours less than 3.5 mm in diameter [83]. Furthermore, cryotherapy is contraindicated when the tumour diameter exceeds 3.5 mm. The therapy involves using a needle-like applicator called a cryoprobe and liquid nitrogen or argon gas. The cryoprobe is placed in close proximity to the tumour, either directly on the sclera or the conjunctiva and liquid nitrogen is released. The triple freeze-thaw technique is then employed to facilitate the freezing of tumour cells.

*Challenges:* However, in cases other than the presence of vitreous seeds, cryotherapy alone may not be sufficient to eradicate them, and it may need to be combined with systemic chemotherapy or radiotherapy. Furthermore, the vitreous seeds hinder the successful eradication of the tumour cells, and therefore, cryotherapy is inappropriate for such cases [84].

#### 6.2.2 Plaque brachytherapy

Plaque brachytherapy is a treatment approach that involves implanting radioactive material into the sclera over the base where the tumour is located. This method is used to destroy the tumoural cells by irradiating them. The irradiation causes DNA damage, resulting in the death of tumour cells. The implant usually remains intact for 2–4 days, depending on the dose and type of radiation administered, before it is removed [85].

Radioactive materials such as iodine-125 (I) and ruthenium-106 (Ru) are commonly used to treat RB. Among these isotopes, I-125 is the most suitable due to its significant properties, *i.e.*, adequate dose distribution and flexibility of lead shielding. When employing this procedure with I-125, surgeons receive minimal exposure, and it also has little effect on the opposite side of the eye [86]. This therapy is usually considered for tumours that exceed 3 mm in size. Brachytherapy has proven to be effective in reducing tumours up to 16 mm in diameter, and it has a localized effect, resulting in a lower risk of radiation-induced secondary carcinoma. Even better results are achieved when it is used in combination with IAC [87].

*Challenges:* Long-term exposure to brachytherapy possesses adverse effects, leading to secondary tumours and cataracts.

#### 6.2.3 Thermotherapy

This treatment modality employs a diode laser emitting a wavelength of 810 nm, which generates heat ranging from 42 to 60 °C, to instigate a cytotoxic effect on tumour cells. This temperature range is selected to be just below the threshold temperature that could lead to coagulation, thereby avoiding damage to the retinal vessels from coagulation [88]. When combined with chemotherapy, thermotherapy can be used to treat tumours less than 3 mm in diameter without the presence of vitreous seeds [89,90]. Indocyanine green (ICG) is used to enhance the effect of thermotherapy in cases when tumours are either less sensitive or unresponsive to conventional thermotherapy. However, it has been reported that the chances of regression are comparatively lower when ICG is used in conjunction with thermotherapy [90].

*Challenges:* It is unsuitable for large tumours and can damage the surrounding healthy tissues.

#### 6.2.4 Laser photocoagulation

It successfully eradicates tumours that are less than 3 mm in diameter and confined to the retina [91]. A 520 nm argon laser is most commonly used, followed by a diode laser or a xenon arc. The laser generates heat of more than 65 °C, which effectively coagulates the blood circulation around the tumour. It is important to note that laser therapy should typically be performed no more than 24 h after intravenous chemotherapy with carboplatin, as it helps to enhance the efficacy of the treatment. It is worth noting that the laser does not target the tumour tissue directly but rather coagulates the blood circulation around the tumour, leading to its subsequent eradication [92].

#### 6.2.5 External beam radiotherapy

This therapy is a viable treatment option for RB cases that are resistant to other focal therapies. A linear accelerator delivers High-energy radiation to the tumour site via electron and photon beams. The commonly prescribed dose is 45 Gy and is administered in fractions of 1.8 Gy over a prolonged period of 4–8 weeks [93]. It has proven extremely useful in treating multifocal RB and vitreous seeds in large tumours unresponsive to all other treatment modalities. The incidence of tumour recurrence following external bean radiotherapy is contingent upon the tumour severity at the time of treatment administration [94].

### 6.3 Enucleation

It corresponds to the surgical removal of the affected eye and a long optic nerve segment to prevent seeding and extraocular spread. After removing the eye, an artificial implant is inserted to restore the orbit volume. This treatment modality is generally preferred for unilateral RB when the tumour is large, bleeding, and spreading near the front of the eye or when the tumour no longer responds to various chemotherapies [95].

## 7 Novel drug delivery strategies

Traditionally, several treatment modalities have been used to treat RB, including chemotherapy, radiotherapy, and enucleation. However, these treatments have been associated with side effects such as dry eye, secondary cancers, and renal toxicity [96]. Several new drug delivery systems have been developed to overcome these limitations, offering improved RB treatment solutions with fewer adverse effects. These novel therapies can increase drug retention time and help to overcome physiological defence barriers [97].

### 7.1 Nanostructured drug delivery systems

**Fig. 3:**
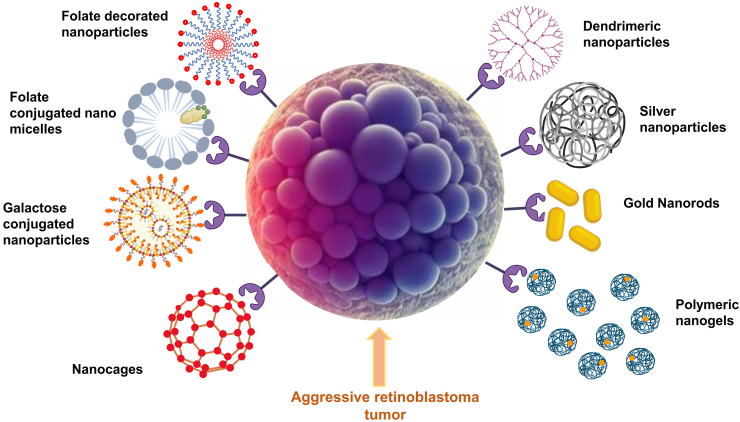
Novel therapies employed for the treatment of retinoblastoma.

Nanostructured drug delivery systems have emerged as a promising solution for treating RB, owing to their ability to sustainably release antineoplastic drugs and their potential for surface modification with various ligands that can enhance tumour tissue targeting. These systems play a critical role in reducing medication toxicity, a major concern in treating RB. Several types of drug delivery systems, such as ligand-conjugated nanoparticles [[98], [99], [100], [101], [102], [103], [104]]^]^, polymeric nanoparticles [[105], [106], [107], [108], [109], [110], [111]], metallic nanoparticles [[112], [113], [114]], nanoliposomes [115,116], nano-micelles [117], micelles loaded thermosensitive gel [118], polymeric micelles [119], polymeric nanogels [120], hydrogels [121], gold conjugated nanoparticles [122], niosomes [123], liposomes [124] etc., have been reported for the treatment of RB (Table 2 and Fig. 3).

**Table 2:** Various reported drug delivery systems for retinoblastoma (RB).

Drug Delivery System	Drug incorporated	Route of administration	Formulation Composition	Key Findings	References
Mesoporous silica nanoparticles	Carboplatin	*In vitro* study on Y79 cell line	N-Cetyltrimethylammonium bromide, Tetraethylorthosilicate, sodium hydroxide (NaOH)	• Improved internalization was observed upon conjugation with EpCAM due to targeting specific receptors. • The IC50 value of free Carboplatin was higher than that of the EpCAM-conjugated nanoparticles, indicating an enhanced anticancer effect.	[98]
Folate decorated nanomicelles	Curcumin-difluorinated	*In vitro* study on Y79 and WERI cell line	Poly (styrene-*co*-maleic anhydride) (average MWt 1600), N-(3-(dimethylamino) propyl)-N-ethylcarbodiimide hydrochloride (EDC)	• Conjugation with folic acid aided in pronounced cell death in both Y-79 and WERI RB cell lines. • Retinal pigment epithelial cells showed no cytotoxicity signs, indicating the formulation's safety.	[99]
Folate decorated nanoparticles	Doxorubicin	*In vitro* study on Y79 cell line	Chitosan, Sodium triphosphate pentabasic	• Higher intracellular uptake of conjugated nanoparticles had a significant impact on the cytotoxic effects of the formulation. • Folate decoration activated downstream caspases for apoptotic activity.	[100]
Galactose conjugated nanoparticles	Etopsoside	*In vitro* study on Y79 cell line	Resomer RG PLGA 502H, Chitosan, EDC, N-hydroxysuccinimide (NHS)	• Sugar receptors in Y-79 cells were actively targeted by the conjugated nanoparticles. • The tumour cells' cellular uptake of conjugated nanoparticles spiked more than the non-conjugated ones. • Pronounced apoptotic activity of conjugated nanoparticles.	[101]
Ceria Nanoparticles	Doxorubicin	Subretinal	AMD11070 (C-X-C Chemokine receptor 4 Antagonist), BAC (N–N' -bis acrolyl cystamine) (triblock terpolymers), glycol chitosan	• Effective and precise targeting of tumour cells. • Chemokine receptor antagonist diminishes the off-target effects of native drug incorporated, strengthening the therapeutic efficacy.	[102]
Cerium doped Nanoparticles	Titanium dioxide	*In vitro* study on Y79 cell line	Cerium nitrate hexahydrate	• The Ce-doped nanoparticles possessed improved cytotoxicity, thus depicting enhanced anticancer activity. • Upon *UV* radiation, the cancer cells with doped nanoparticles decreased cell viability, thereby improving survivability.	[104]
Nanoparticles	Carboplatin	Subconjunctival	Half-generation poly (amidoamine) dendrimer (G3.5 PAMAM)	• High-dose carboplatin in mice reduced the tumour mass significantly more than conventional carboplatin. • Higher dose injections created no toxicity and were greatly more effective than the carboplatin solution.	[106]
Folate decorated nanoparticles	Nutlin-3a and curcumin	*In vitro* study on Y79 cell line	Poly (D, l-lactide-*co*-glycolide) PLGA, N-hydroxysulfosuccinamide (Sulfo-NHS), 1, 3, Dicyclohexyl carbodiimide, polyvinyl alcohol	• The therapeutic efficacy of dual drug-loaded nanoparticles was greater than the other formulations. • Curcumin augmented the efficacy of nutlin-3a by modulating Multi-Drug Resistance (MDR).	[107]
Surface modified nanoparticles	Melphalan	Intravitreal	Carboxyl-terminated poly(lactic-*co*-glycolic acid; PLGA), polyvinyl alcohol	• Surface modification levelled up the therapeutic efficacy of melphalan nanoparticles compared to the unmodified nanoparticles. • Enhanced cell internalization was observed.	[108]
Polymeric nanoparticles	Paclitaxel	Intravenous	PLGA, ethyl-3-[3-dimethylaminopropyl] carbodiimide hydrochloride, N- hydroxy-succinimide, EpCAM (Epithelial Cell Adhesion Molecule)- FITC (Fluorescein Isothiocyanate) antibody	• Polyethylene glycol (PEG) coating improved the biocompatibility of the formulation. • Cellular uptake hiked in the case of chitosan-coated nanoparticles. • Prolonged drug retention time was achieved. • Also, the formulation had higher cytotoxicity.	[109]
Nanoparticles	Carboplatin and Etoposide	*In vitro* study on Y79 cell line	3-(4,5-Dimethylthiazol-2-yl)-2,5-diphenyl-tetrazolium bromide.	• The cytotoxicity levels of the dual drug-loaded conjugated nanoparticles displayed a significant increment compared to the standard drug treatment. • Drug uptake improved when lactoferrin was used for conjugation.	[110]
Nanoparticles	Doxorubicin hydrochloride and vinblastine	Subconjunctival	PLGA, Human Serum Albumin	• Sustained release obtained by depot mechanism. • The drug diffused faintly from the formulation as compared to the native drug.	[111]
Gold Nanoparticles	–	*In vitro* study on Y79 cell line	control peptide (KRLRLDPV, 8 amino acids)	• Delivering HDM2 (human double minute) upregulated the p53 protein which is necessary for controlled cell growth. • HDM2-loaded Au nanoparticles affected apoptotic activity positively by G2M phase cell cycle arrest. • Apoptosis was mediated by inducing p53-inducible miRNAs.	[112]
Gold nanoparticles	Rosiglitazone	*In vitro* study on Y79 cell line	–	• Rosiglitazone-loaded nanoparticles worked by blocking the activation of the PI3K/Akt signalling pathway. • The antiproliferative effect and the apoptotic activity were observed to be more pronounced.	[113]
Silver Nanoparticles	Laminarin	*In vitro* study on Y79 cell line	Brown seaweed Turbinaria Ornata, Silver nitrate	• Cell viability was successfully reduced in a dose-dependent manner. • Significant apoptotic activity. • Elevated inhibition of Y79 cell lines on successful conjugation with laminarin.	[114]
Super-magnetic liposomes	Indocyanine Green	*In vitro* study on Y79 cell line	DPPC (1,2-Dihexadecanoyl-rac-Glycero-3-Phosphocholine), DSPE-PEG (2000)-FA (1,2-distearoyl-*sn*-glyc-ero-3-phosphoethanolamine-N-[folate(polyethylene glycol)-2000]) and Superparamagnetic iron oxide nanoparticles (SPION	• Folate conjugation had a major impact on the targeting of the nanoparticles, with a high cell uptake rate of 95 %. • Y79 cell study showed that the liposomes had an augmented anticancer effect on them, with complete tumour regression. • The photothermal/photodynamic therapy synergistically downregulated the HIF-1 α and HSP70 expression.	[115]
Nanoliposomes	Melphalan	Intravitreal	Dipalmitoyl phosphatidylcholine (DPPC), Xylazine, Ketamine	• Drugs leaked from the lipid bilayer of liposomes, and immediate drug degradation occurred in vitreous media. • Dosing more than 10 mg created retinal toxicity.	[116]
Folate micelles loaded thermosensitive gel	Doxorubicin	Intravenous	Poly-oxy-ethylene bis (amine), folic acid, dicyclo-hexyl-carbodiimide, triethylamine, Poly (lactide-*co*-glycolide) PLGA-PEG-PLGA	• Exhibited higher cytotoxicity in Y-79 cells. • Sustained drug release for up to 2 weeks. • Higher cellular uptake of Doxorubicin micelles.	[118]
Polymeric micelles	Apigenin	Intravenous	Pluronic F68, Pluronic F127, Pluronic 123, PEG (2000)- PLA	• Initial burst release followed by a sustained release. • Effective in the reduction of tumour cell growth.	[119]
Polymeric nanogels	Voronistat and Etoposide	Intravenous	Oligo (ethylene glycol) monomethyl ether methacrylate, poly(ethylene glycol) mono-methacrylate, mono-methyl-ether, Bromo-2-methyl propionic acid, 3,3-dithiopropionic acid, copper bromide, Span 80, l-ascorbic acid	• A synergistic effect was observed compared to single-encapsulated or free drugs. • Improved cell apoptosis of etoposide when combined with vorinostat.	[120]
Hydrogels	Topotecan hydrochloride	Intra-vitreal	PEGs, poly ε-caprolactone, tin (II) 2-ethyl-hexanoate	• Cytotoxicity observed towards RB cells. • Sustained release for up to a week. • Anti-proliferative activity was observed with greater drug loading.	[121]
Nanoparticles	Gold Anti-EpCAM (Epithelial Cell Adhesion Molecule) conjugated loaded with siRNA	*In vitro* study on Y79 cell line	Tetrakis-hydroxymethyl-phosphonium chloride, branched polyethyleneimine, 6-fluorescein amidite-siRNA.	• Gold nanoparticles loaded with siRNA had an effect twice that of the naked siRNA. • EpCAM conjugation would specifically target the tumour cells; thus, surrounding cells remain unaffected.	[122]
Niosomes	Hyaluronic acid	Intravitreal	Squalene, 1,2-dioleoyl-3-trimethylammonium-propane (DOTAP), 1,2-dioleyl-*sn*-glycero-3-phosphoethanolamine (DOPE)	• Negligible cytotoxicity levels were observed with 90 % cell viability. • Strong induction of Enhanced Green Fluorescent protein (EGFP) expression depicting good cell penetration.	[123]
Liposomes	Triamcinolone acetonide	–	Soybean phosphatidylcholine, chitosan, coumarin-6, cholesterol	• The prolonged and sustained release was observed without an initial burst release. • Possessed better potency for drug delivery.	[124]
Nanoparticles	Topotecan	Intravitreal	Low-molecular-weight chitosan (Cs), sodium tripolyphosphate (TPP), EDC, NHS	• Modifying nanoparticles with chitosan led to the upgraded therapeutic efficacy of the nanoparticles as compared to unmodified ones. • More significant tumour necrosis was observed in the case of modified nanoparticles.	[127]

#### 7.1.1 Ligand-conjugated nanoparticles

Nanoparticles loaded with drugs can be conjugated with biodegradable ligands to target receptors overexpressed in RB, such as receptors for folic acid, hyaluronic acid, and galactose [125].

Folate receptors, in particular, are overexpressed in RB cells compared to normal cells [126]. Hence, the surface conjugation of folic acid with nanoparticles or other drug delivery systems can serve as an effective therapeutic approach for treating this disease.

Delrish et al. [127] demonstrated an improvement in the therapeutic efficacy of topotecan by conjugating nanoparticles with folic acid. Through folate conjugation, mesoporous silica nanoparticles were found to be more effectively taken up by RB cells than those without folic acid decoration. This increased cellular uptake resulted in an enhanced cytotoxic effect compared to other formulations. Furthermore, the presence of folic acid on the surface of the nanoparticles helped to control the release of the drug. The improved targeting capability of the nanoparticle formulation was also shown to lead to a greater reduction in the tumour volume, as observed after *in vivo* studies.

In their study, Alsab et al. [99] utilized folic acid to target curcumin-difluorinated (CDF)-loaded nano micelles. The results clearly showed that folic acid increased the cytotoxic property of CDF. Moreover, folate conjugation led to a decreased IC_50_ value. Parveen et al. [100] developed folate-decorated nanoparticles loaded with doxorubicin, showing a more substantial antiproliferative effect on tumour cells than doxorubicin nanoparticles alone. The folate conjugation facilitated a higher cytotoxic effect, and a lower concentration was required for 50 % tumour cell killing.

Sugar receptors are expressed on RB cells. Polymeric nanoparticles can be conjugated with galactose and mannose residues to target drug molecules to these receptors selectively. This strategy allows the sugar moieties to serve as preferred ligands for the targeted delivery of drugs to the cancer cells. Furthermore, conjugating these sugar residues to the polymeric nanoparticles can enhance drug loading capabilities, resulting in more effective drug delivery to the RB cells [101].

Godse et al. [101] conjugated etoposide nanoparticles with galactose, and the study showed a significant increase in the cellular uptake of galactose-decorated nanoparticles. The apoptotic activity of the conjugated preparation was found to be potentially higher compared to the non-conjugated nanoparticles.

Gao et al. [102] utilized nanoceramics to conjugate with doxorubicin nanoparticles with a C-X-C chemokine receptor four antagonists. The conjugation of the chemokine receptor resulted in a significantly effective targeting system, reducing the off-target effects of doxorubicin and ultimately enhancing the therapeutic efficacy of the entire formulation.

#### 7.1.2 Polymeric nanoparticles

Nanoparticles are modified by natural or synthetic polymers to enhance the pharmacokinetic properties, stability, and anticancer efficacy of drugs while reducing their toxicity. In the context of RB, commonly used polymers include poly (lactic-*co*-glycolic acid) [PLGA], polyamidoamine [PAMAM], polyethyleneglycol [PEG], chitosan and polycaprolactone [128]. Kang et al. [106] studied the PAMAM dendrimer nanoparticles loaded with carboplatin and found they could easily reach the intraocular tissue due to their small size. Moreover, nanoparticles larger than 200 nm were retained in the subconjunctival region for an extended period.

Das et al. [107] investigated the effectiveness of PLGA nanoparticles loaded with curcumin and nutlin-3a, and conjugated with folate for targeting RB cells. The conjugation of folate on the surface of the nanoparticles levelled up the cellular uptake, and the dual drug loading resulted in enhanced antiproliferative activity. This research study indicated that combining two drugs with specific target cells in a single formulation exhibited a synergistic effect, ultimately leading to increased apoptotic activity.

Sims et al. [108] modified the surface of melphalan-loaded PLGA nanoparticles. The study revealed a substantial increase in the drug loading capacity of the nanoparticles, up to 85-fold, after surface modification with PLGA and polyvinyl alcohol (PVA). The sustained release of melphalan obtained was directly proportional to the PVA solutions used for saturation. This innovative approach obviates the need for regular intravitreal administration, offering a promising solution for ocular chemotherapy [108].

Qu et al. [98] used PLGA and sodium alginate for surface modification of carboplatin-loaded nanoparticles. The results demonstrated that adding sodium alginate significantly affected carboplatin release and exhibited a lower percentage of release bursts compared to nanoparticles modified with PLGA alone. This indicates that sodium alginate contributed to a sustained release of the formulation. The inhibitory effect of the nanoparticles was also observed to be higher with the addition of sodium alginate, which was attributed to greater cellular uptake. Also, cell line studies have shown a clear improvement in the penetration of sodium alginate nanoparticles [98].

Delrish et al. [129] developed and studied the therapeutic efficacy of thiolated chitosan-dextran nanoparticles loaded with topotecan through intravitreal administration for RB in a rabbit xenograft model and Y79 human RB. Trimethyl chitosan (TMC) is used owing to its mucoadhesive potential. Carboxymethyl Dextran (CMD)- TMC cysteine conjugated (TCs) topotecan loaded nanoparticles contributed in two ways: first, both CMD and TCs combinedly resolved the stability constraint of topotecan. Secondly, the CMD surface stabilized the topotecan nanoparticles. This phenomenon obstructed the agglomeration of nanoparticles right after intravitreal administration. The tumour necrosis percentage indicated that topotecan-loaded nanoparticles were much more efficacious than topotecan in rabbits. Also, the tumour volume significantly reduced after treatment with the conjugated nanoparticles. The IC_50_ value of conjugated nanoparticles was found to be lower than the free topotecan in Y79 human RB cells.

#### 7.1.3 Metallic nanoparticles

The therapeutic efficacy of anticancer drugs can be enhanced by metallic nanoparticles due to their high drug loading capacity, photothermal behaviour, and ability to accurately control electrostatic charge, size, shape, and surface modification [130].

Gold (Au) nanoparticles were formulated by Kalmodia et al. [112] for the delivery of anti-HDM2 (human double minute) peptides. Before treatment, wild-type p53 was found to be strongly downregulated in the RB cell line, but the use of Au nanoparticles upregulated the p53 protein by interfering with the ubiquitination-mediated proteolysis of the protein's expression. *In vitro,* studies have demonstrated that Au nanoparticles can induce apoptosis by blocking the G2M phase of the cell cycle [112].

Yao et al. [113] prepared gold nanoparticles loaded with rosiglitazone and investigated their therapeutic efficacy in the *RB1* cell line. They observed a significant decrease in the proliferation rate of the tumour cells and an apoptotic effect. Phosphoinositide 3-kinase inhibitors were used to make the effect of nanoparticles on the P13K/Akt pathway more pronounced. The formulation inhibited the P13/Akt signalling pathway, negatively affecting cell proliferation [113].

Remya et al. [114] synthesized silver (Ag) nanoparticles loaded with laminarin isolated from *Turbinaria ornata*. These Ag nanoparticles were further evaluated for their apoptotic activity on Y79 cell lines. Their findings revealed that the cytotoxicity of the nanoparticles was found to be directly proportional to the dose. Interestingly, the laminarin-conjugated Ag nanoparticles exhibited a significant reduction in the rate of Y79 cell proliferation, with an IC50 value of 10.5 μg/mL. Furthermore, the study highlighted the synergistic effects of laminarin as a capping agent for the Ag nanoparticle. The result of the study suggests that laminarin-conjugated Ag nanoparticles have great potential as an effective therapeutic agent for the treatment of RB [114].

#### 7.1.4 Nanoliposomes

Zheng et al. [115] developed a highly innovative therapeutic formulation, namely folate superparamagnetic dual-targeted cationic nanoliposomes loaded with indocyanine green and perfluorohexane (FCNPIFE), to achieve synergistic photothermal and photodynamic therapy of RB. Folate and magnetic decoration enabled a substantial nanoliposome invasion into the tumour area. The nanoliposomes displayed pronounced chemotherapeutic efficacy against Y79 cells. In addition, the targeted photothermal and photodynamic effects were achieved by downregulating the expression of *HIF-1a* and *HSP70,* leading to complete regression of the tumour [115].

#### 7.1.5 Nanomicelles

Curcumin-difluorinated (CDF)-loaded folate-targeted nanomicelles were synthesized by Alsaab et al. [99] The safety of the nanomicelles was demonstrated by the absence of cytotoxicity in Y79 and WERI-RB cells. Strong apoptotic activity was observed in both cell lines, indicating their potential as a therapeutic option for RB. In addition, folic acid conjugated with styrene-co-maleic acid significantly altered the properties of CDF, inducing cell death [99].Synthesis of controlled-release tumour-targeted celastrol nanomicelles and their delivery by reduction-sensitive nanomedicine benefited RB treatment [117].The apoptotic potential of celastrol had not been previously investigated. The study of nanomicelles on Y79 cells demonstrated the cytotoxic activity of celastrol, which resulted in the inhibition of uncontrolled cell proliferation. Furthermore, the study also elucidated the apoptotic mechanism, revealing an activation of caspases (*caspase-3* and *caspase-9*) [131].

#### 7.1.6 Nanocages

A nanocage loaded with abemaciclib and IMD 0354, synthesized by Yang et al. [132], inhibited the expression of cyclin D through the specific use of a charge reversal polymer. Triple combination therapy arrested the cell cycle in the G1 phase by abemaciclib, and cyclin D levels were decreased by IMD 0354. Abemaciclib selectively inhibited the uncontrolled proliferation of T cells. The synergistic effect attenuated the chemotherapeutic action of abemaciclib and IMD 0354 [132]. Gold nanocages exhibit several unique features that make them suitable for targeted therapeutic applications. One of their distinctive features is the porous walls, which lend themselves to the controlled delivery of drugs [133].

## 8 Next generation technologies

In the pursuit of effective treatment for RB, both conventional treatment strategies and novel drug delivery systems have been explored. However, molecular targeting is an even more promising approach, as it acts directly on the signalling pathways responsible for tumour growth. For example, the MDM2 protein negatively regulates the p53 pathway, and when the former is overexpressed, it acts as an oncogene [53]. As a result, the activity of p53 to regulate cell growth is impaired by this overexpression of MDM2 protein [134]. In-depth knowledge of these pathways that trigger tumour growth has led to advances in treating RB [135].

### 8.1 Novel drug molecules and targets

Drug molecules that may show promise are outlined below.

#### 8.1.1 Nutlin-3a

This drug molecule inhibits the interaction of MDM2 and MDMX proteins with the p53 pathway, thereby suppressing tumour growth. When the MDM2 and MDMX interaction is hindered, the normal function of p53 is restored, leading to successful control of cell growth and proliferation [136]. Elison et al. [137] conducted clinical studies on the therapeutic efficacy of Nutlin-3a against Y79 cells. This small molecule inhibitor successfully led to significant apoptotic activity and cell death. Nutlin-3a is undergoing a phase-1 clinical trial, and positive outcomes are expected.

#### 8.1.2 Pentoxifylline

This phosphodiesterase inhibitor has significantly improved apoptotic activity in Y79 RB cells. When combined with carboplatin, pentoxifylline restricts IĸBα phosphorylation and inhibits NF-ĸB activity. This combination therapy results in upregulating *caspases -3, -8, -9, Bak, Bad,* and *Bax*, known proapoptotic genes [138].

#### 8.1.3 Ribavirin

It selectively targets the function of eIF4E, which is known to play a pivotal role in the growth and development of cancerous tumour cells. Additionally, Ribavirin has been shown to slow down the process of angiogenesis effectively. Notably, this frug molecule only blocks the functions of eIF4E, c-MYC, and VEGF without aiming to reduce their concentrations in the body [139].

#### 8.1.4 EDL-155

The isoquinoline derivative, EDL-155, has shown some efficacy in preclinical studies; however, its effect on the Y79 cell line was weak. It acts on average tumour burden by inhibiting mitochondrial action on cancer cells without causing significant systemic toxicity [140].

#### 8.1.5 HDAC inhibitors (histone deacetylase inhibitors)

HDAC inhibitors represent a promising new category of antineoplastic agents with the potential to induce cytotoxicity selectively [141]. Dalgard et al. [141] conducted Preclinical studies showing that HDAC inhibitors exert strong apoptotic activity on RB cell lines in murine models. Since the effect of this modality was specific and selective, it was concluded that HDAC inhibitors would exhibit less or no systemic toxicity in clinical use.

#### 8.1.6 N-MYC inhibitors

The MYC protein, which is oncogenic in nature, plays a crucial role in regulating various cellular mechanisms such as cell growth, proliferation, and apoptosis. Research by Lee et al. [142] has shown that this protein is multiplied and overexpressed in RB. Therefore, targeting this protein could be a potential approach to treat RB [142]. Sradhanjali et al. [143] showed that targeting N-MYC increased apoptosis in Y79 cells. This was attributed to the induction of the p53 signalling pathway. Another promising strategy is the combined use of the chemotherapeutic agent carboplatin and N-MYC inhibitors (10058-F4) against RB cell lines. This approach exhibited a synergistic effect with a massive jump in the inhibition of cell proliferation. This approach needs to be further refined to treat RB effectively.

#### 8.1.7 SYK inhibitors

The overexpression of SYK (spleen tyrosine kinase) and its role in promoting aggressive cell division makes it a critical target for treating RB. SYK inhibitors suppress MCL-1, which belongs to the BCL2 class, leading to the silencing of SYK proto-oncogene. Surprisingly, it is not expressed in the normal retina, making it an important target for treating RB [144,145].

#### 8.1.8 Matrix metalo proteinase (MMP-2) and MMP-9

Extracellular matrix degradation, which is thought to play a key role in metastasis, is primarily mediated by matrixes, a class of zinc-dependent proteins that include MMP-2 and MMP-9 [146]. Therefore, targeting these proteins could potentially reduce metastasis. In a study by Webb et al. [147], the angiogenic response of Y79 was found to be decreased by the MMP-9 inhibitor AG-L-66085, suggesting its potential use as an additional therapy.

#### 8.1.9 Galenic preparations

In this type of preparation, named after Claudius Galen, biodegradable polymers are combined with a therapeutically active molecule of either synthetic or natural origin. The primary goal of these Galenic formulations is to improve the pharmacokinetic properties of the drug. Additionally, small antineoplastic agents exhibiting heat-reactive characteristics may be considered an alternative to hyperthermia treatment [24].

### 8.2 Immunotherapies

**Fig. 4:**
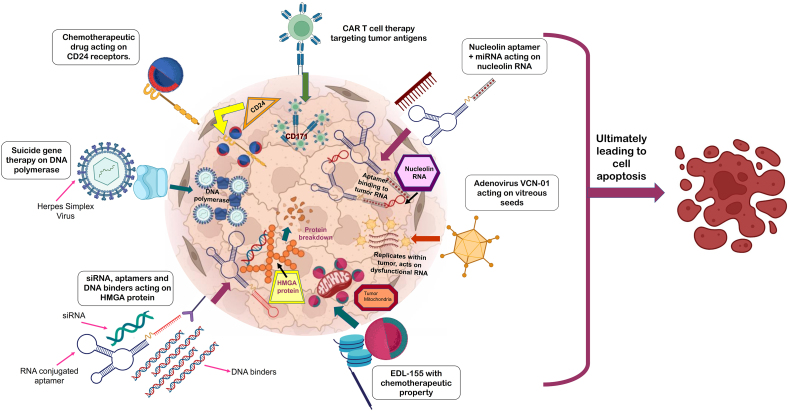
Next-generation management strategies with their potential targets (Starting clockwise is CAR T-cell therapy, in which CAR T-cells potentially target antigens (CD171) on retinal tumour cells and induce cytotoxicity. Locked nucleic acid-modified nucleolin aptamer strongly inhibited tumour growth. Adenovirus VCN-01 acted on the modified RNA and inhibited cell proliferation. EDL-155, an isoquinolone derivative, targeted tumour mitochondria to prevent further uncontrolled cell division. HMGA protein overexpression in RB is antagonized by RNAa-conjugated aptamer, siRNA, and DNA binders, which bind to the protein to cause its degradation. Suicide gene therapy uses the herpes simplex virus to act on DNA polymerase, inhibiting mutant DNA catalysis. The CD24 receptors are targeted by vincristine VCR to produce an apoptotic effect on the tumour cell).

Conventional treatment options have certain shortcomings, so new therapeutic alternatives have emerged. The combination of molecular targeting with immunotherapy approaches has opened up new horizons for tumour eradication therapy. By selectively targeting specific receptors, immunotherapies offer a promising alternative mode of treatment [148] (see Fig. 4).

#### 8.2.1 GD2 specific chimeric antigen receptor (CAR)-modified T-cell therapy

GD2 is a disialoganglioside highly expressed in RB cells, making it a potent target for RB. The CAR T-cells have been genetically modified to produce chimeric proteins on their surface, allowing them to target and destroy tumour cells specifically [149]. This CAR T-cell therapy may be beneficial in metastatic RB. CAR T-cells potentially target the CD171 antigens on retinal tumour cells, inducing cytotoxicity. Sequential modification of antigens in cell therapy successfully eradicates RB cells. Future studies of this therapy, *in vivo* and in vitro, would be of great interest in the early treatment of this disease [150].

#### 8.2.2 Nucleolin protein

The nucleolin (NCL) protein expression is markedly higher in retinal tumour cells than in normal ones. Being a nucleolar RNA, it is responsible for carcinogenesis. The NCL aptamer can effectively decrease the proliferation rate in tumour cells as well as tumour miRNA-18a and serum levels of miRNA-18a. Furthermore, nude mice strongly inhibited tumour growth by modifying the NCL aptamer with a locked nucleic acid [151].

#### 8.2.3 Signal transducer CD24

The high expression of the differentiation 24 (CD24) cluster, a cell surface receptor, in RB cells makes it another potential target for treating retinal tumours. One of the chemotherapeutic agents that have been used to target CD24 is vincristine (VCR), which acts on the PTEN/Akt/mTORC1 pathway to selectively inhibit CD24 activity [152]. This approach has shown promise in preclinical studies and could be a potential therapy for RB in the future.

### 8.3 Gene therapies

Gene therapies consist of introducing genetically engineered cells to save the affected eye(s) by reducing the tumour burden. Several studies have been conducted on delivering genes to the tumour site, offering a promising approach to treating RB [153].

#### 8.3.1 Herpes simplex virus tyrosine kinase (HSV/Tk) gene

It is also known as suicide gene therapy, in which the HSV/Tk gene is introduced intravitreally along with ganciclovir (GCV). This approach benefits from the synergistic effects of inhibiting DNA polymerase. The monophosphorylation of ganciclovir is triggered by the production of proteins resulting from the modification of RB cells and linked to the action of *HSV/Tk.* [154] Successful transduction by an adenoviral vector was observed. The treatment results exhibited a visible reduction in tumour size after one week of therapy. A decrease in vitreous seed size was also observed in the immunodeficient mice. This therapy may be considered an effective treatment option up to the stage of vitreous seed development and may also be used in conjunction with other chemotherapies [155].

#### 8.3.2 RB gene fragments

RB gene therapy is considered a promising approach, utilizing RB gene fragments with high potency to suppress tumour cells. Studies have shown that the RB protein, with a truncated N-terminus of 94 kDa (pRB94), can strongly suppress tumour cells in their non-phosphorylated form. This form allows pRB94 to interact with E2F, a transcription factor that regulates cell proliferation. When pRB94 reaches the tumour site, it prevents tumour cells from entering the S phase of their cell cycle, eventually leading to apoptosis. Using recombinant adenovirus vectors to introduce pRB94 at the preclinical level has yielded positive results, with most retinal tumours halting growth and others shrinking significantly. Thus, pRB94 can be a highly effective antiproliferative agent in treating RB [156].

#### 8.3.3 Adenovirus VCN-01

VCN*-*01 is a genetically engineered clinical adenovirus with oncolytic properties that have explicitly been modified to inhibit the aggressive proliferation of cancer cells in the retina [157]. During the initial phase of preclinical studies, it was observed that the adenovirus remained localized in the retinal area with minimal leakage. In the phase-2 clinical trial, it was found that the tumour burden decreased significantly, and there was a marked reduction in vitreous seeding after the first dose. Thus, this may represent another treatment alternative to preserve vision to some extent [158].

#### 8.3.4 HMGA protein

Overexpression of the HMGA protein in RB makes it another potential target for therapy. Inhibiting the proliferative activity of tumour cells can be achieved using netropsin, DNA minor groove binders, aptamers, and siRNAs that specifically target the HMGA protein. Targeting is best mediated by an NCL-decorated antibody containing HMGA aptamers and exhibiting decreased cytotoxicity, as observed in WERI-*RB1* cell lines [159].

#### 8.3.5 Long non-coding RNAs (lncRNAs)

These RNAs play a role in regulating gene expression and have been found to contribute to RB progression by inhibiting apoptosis and promoting cell proliferation through the activation of the P13K/Akt pathway. For the induction of apoptotic activity, the LncRNA taurine-upregulated gene 1 (TUG1) was one of the most frequently found oncogenes in RB. Deleting these long non-coding RNAs would be beneficial in inducing apoptotic activity and regulating cell proliferation [160].

#### 8.3.6 Circular RNAs

Circular RNAs (circRNAs) have emerged as a new class of non-coding RNAs that play a crucial role in regulating gene expression. Silencing of Circ-FAM158A strongly affects cell apoptosis by limiting uncontrolled cell proliferation in vitro and *in vivo*. The miR-138-5p-dependent regulation of pater33nally expressed gene 10 (*PEG10*) is the most common pathway for circRNAs, which is involved in RB progression [161].

## 9 Clinical trials

The treatment of RB has always been challenging and life-threatening, and efforts are being made worldwide to achieve the best results through various treatment strategies. So far, 125 clinical trials have been undertaken on RB, as reported in the literature [162]. These trials cover various aspects of the disease, i.e., diagnosis and treatment. Out of these, 32 clinical trials focus on treating RB using different chemotherapeutic agents alone or in combination with other agents and therapies [[163], [164], [165], [166], [167], [168], [169], [170], [171], [172], [173], [174], [175], [176], [177], [178], [179], [180], [181], [182], [183], [184], [185], [186], [187], [188], [189], [190], [191], [192], [193], [194]]. Further, out of 32 clinical studies related to RB treatment, only one clinical trial completed phase 4 [174], and three completed phase 3 [[171], [172], [173]]. However, five clinical trials related to chemotherapeutic agents were terminated [[191], [192], [193], [194]] at various phases, i.e., early phase 1, phase 1, and phase 2. The 32 clinical trials mentioned are listed in Table 3. This table indicates that clinical trials essentially focus on combination therapies with the least invasive method to achieve good therapeutic efficacy. In some clinical trials, focal therapies were used as initial treatment before administering chemotherapies. This approach may be beneficial as it targets specific areas of the retina and reduces the overall dose of chemotherapy required, thus minimizing potential side effects. In most clinical trials, combination therapies have been extensively studied for their effects. A maximum number of clinical trials have been conducted on melphalan [163,164,177,181,182,185,188,189]. However, some trials which employed melphalan individually ^[191-194^ and in combination therapy with carboplatin, etoposide, and vincristine [195] were terminated due to unavoidable adverse effects. The clinical trial that utilized periocular administration of carboplatin, etoposide, and vincristine in combination for six months completed the phase-4 trial and was found to be effective in eradicating nonmetastatic extraocular RB [174].

**Table 3:** Various clinical studies for the treatment of RB.

Drug (s)/Formulations	Study Design	Brief Description	Phase of Trial	Status of Trial	Clinical trial number
Melphalan solution	• Observational case only • Involved 30 participants	• Retrospective review of a 5-year study of super-selective intra-arterial administration of melphalan • The study was conducted in 2 groups, one treated with systemic chemotherapy as first-line therapy and the second group receiving melphalan as first-line treatment.	–	Completed	NCT03935074 [163]
Melphalan solution and Carboplatin solution	• Open-label, single-group assignment • Enrolled 10 participants	• Intra-arterial administration of melphalan and carboplatin was performed in subjects with advanced, recurrent RB.	Not applicable	Completed	NCT00857519 [164]
Carboplatin solution and Maxitrol solution	• Open-label, single-group assignment • Enrolled 8 participants	• Intravitreal administration of carboplatin was performed to study safety and toxicity in recurrent intraocular RB and the vitreous seed.	Phase 1	Completed	NCT02792036 [165]
Topotecan solution	• Open-label, non-randomized single-group assignment • Enrolled 5 participants	• A specific case of recurrence-resistant tumours was considered for dose escalation evaluation using the accelerated titration method. The limit of the dose was higher grade ocular toxicity.	Phase 1	Completed	NCT00460876 [166]
Carboplatin solution, Filgrastim, Cisplatin solution, Cyclophosphamide solution, Etoposide solution, Vincristine sulfate solution	Involved 50 participants	• Combination therapy was studied for intraocular RB to determine survival and time to failure. The solution of carboplatin, vincristine, cisplatin, etoposide, and cyclophosphamide was infused intravenously, and filgrastim was administered subcutaneously. • Finally, the combined toxicity was assessed, and the percentage of healthy eyes after therapy was compared with eyes that had received radiotherapy.	Phase 2	Completed	NCT00002675 [167]
Carboplatin and Vincristine Sulfate solution	• Enrolled 25 participants	• The sole objective was to record the response to carboplatin and vincristine over 24 weeks. • The cumulative incidence function of treatment failure was determined to predict treatment success at 4.5 years.	Phase 2	Completed	NCT00002794 [168]
Carboplatin solution, Cyclophosphamide solution, Doxorubicin hydrochloride solution, Etoposide solution, Topotecan hydrochloride solution	• Enrolled 5 participants	• The efficacy and feasibility of combination therapy of the mentioned drugs before bone marrow transplantation were evaluated in participants with metastatic or extra choroidal RB.	Phase 2	Completed	NCT00004006 [169]
Carboplatin solution	• Open-label, non-randomized, single-group assignment • Enrolled 30 participants	• The combined and individual effects of carboplatin therapy with laser therapy and cryotherapy were evaluated.	Phase 2	Completed	NCT00179920 [170]
Liposomal vincristine sulfate, Carboplatin solution, Etoposide solution	• Open-label, non-randomized, parallel assignment • Enrolled 331 participants	• The study aimed to evaluate the histopathologic features of recently diagnosed individuals with unilateral RB and enucleated eyes. • During the study, choroidal involvement, tumour invasion of the optic nerve, scleral involvement, and anterior segment involvement were observed and reported. • Event-free survival and overall survival were determined in individuals with and without chemotherapy. • Finally, the toxicity profile of chemotherapy was determined.	Phase 3	Completed	NCT00335738 [171]
Liposomal vincristine sulfate, Carboplatin solution, Etoposide solution, and Filgrastim solution	• Open-label, single-group assignment • Enrolled 30 participants	• Systemic chemotherapy of carboplatin, etoposide, subcutaneous filgrastim, and subtenon carboplatin was administered to participants of Group D RB. • The long-term toxic events, treatment failure, and percentage of eyes saved without enucleation were studied.	Phase 3	Completed	NCT00072384 [172]
Carboplatin solution, Vincristine sulfate solution	• Open-label, single-group assignment • Enrolled 28 participants	• Neoadjuvant chemo reduction of carboplatin and vincristine sulfate for group B intraocular RB was performed. • Toxicity was evaluated along with response and event-free survival.	Phase 3	Completed	NCT00079417 [173]
Carboplatin solution, Etoposide solution, Vincristine therapy	• Open-label single-group assignment • Enrolled 26 participants	• Participants were subjected to chemotherapy for six months, along with and without carboplatin administration, particularly in cases of non-metastatic extraocular RB.	Phase 4	Completed	NCT02319486 [174]
Carboplatin solution, Etoposide solution, Cytarabine solution, Vincristine sulfate solution	• Open-label, non-randomized study	• In this study, patients were divided into three groups according to the histology of their unilateral tumour. • Group 1 had no histologic features and was referred for orbital recurrence and metastasis. • Group 2a had an anterior tumour spread and, therefore, was treated with a therapy consisting of intravenous carboplatin, etoposide, and vincristine. • Participants in group 2b had tumour invasion in the optic nerve region and were treated with intravenous carboplatin, vincristine, etoposide, and intrathecal administration of cytarabine. Additionally, orbital radiotherapy was also given.	Not applicable	Active	NCT00360750 [175]
Carboplatin solution	• Double-masked, randomized, parallel assignment • Enrolled 60 participants	• Two doses of subtenonal carboplatin administration were compared in cases of group C and D intraocular RB resistant to primary chemotherapy.	Not applicable	Active	NCT00889018 [176]
Melphalan solution		• The efficacy of intra-arterial administration of melphalan was evaluated in unilateral group D RB to curb the enucleation rate otherwise applicable to group D.	Not applicable	Active	NCT02097134 [177]
Filgrastim solution, Carboplatin solution, Cyclosporine solution, Etoposide solution, Vincristine sulfate solution	• Open-label, single-group assignment • Enrolled 71 participants	• The selection of recent RB-diagnosed individuals was made to evaluate the outcome of the combination therapy. In addition, cyclosporine was intravenously administered before laser and cryotherapy.	Phase 2	Active	NCT00110110 [178]
Carboplatin solution, Cisplatin solution, Cyclophosphamide solution, Etoposide solution, Thiotepa solution, Vincristine sulfate solution	• Open-label, non-randomized single-group assignment • Enrolled 60 participants	• In cases of extraocular RB, response and toxicity were determined after multimodality treatment.	Phase 3	Active	NCT00554788 [179]
Nitroglycerine solution	• Double-masked, randomized, crossover assignment • Involved 36 participants	• A double-blind study was performed with concomitant intravenous nitroglycerin and saline simultaneously. • The effects on cardiac and respiratory health were studied in subjects receiving intra-arterial chemotherapy.	Not applicable	Ongoing	NCT04564521 [180]
Melphalan solution	• Open-label, Single-group assignment • Included 18 participants	• The study focused on the intrathecal administration of melphalan in participants with RB metastatic to the CNS. • Further feasibility and therapeutic efficacy were also evaluated.	Not applicable	Ongoing	NCT04903678 [181]
Melphalan solution	• Single group assignment • Included 5 candidates	• The response, feasibility, and toxicity of melphalan after intra-arterial chemotherapy were analyzed.	Phase 1	Ongoing	NCT04342572 [182]
Topotecan Episcleral Plaque	• Open-label single-group assignment • Enrolled 30 participants	• The subjects' active residual or recurrent intraocular RBs were treated with topotecan chemoplaque. • The adverse effects and toxicity after plaque removal were studied.	Phase 1	Ongoing	NCT04428879 [183]
Adenovirus VCN-01	• Open-label, single-group assignment • Included 13 participants	• The primary objective was to determine the safety and tolerability of intravitreally injected VCN-01. • Determination of the maximum tolerated dose was also expected as an outcome.	Phase 1	Ongoing	NCT03284268 [184]
Study 1: Melphalan solution + Topotecan solution	• Open Label, randomized parallel assignment • Involved 225 participants	• Study 1 assessed the therapeutic efficacy of melphalan and topotecan after intra-arterial chemotherapy in combination compared to melphalan individually.	Phase 2	Ongoing	NCT04681417 [185]
Study 2: Etoposide solution, Carboplatin solution and Vincristine solution	• Open Label, randomized parallel assignment • Involved 225 participants	• Study 2 examined the outcomes of visual ability after intravenous therapy in a minimally invasive interventional study.	Phase 3	Ongoing	NCT04681417 [185]
Etoposide solution, Vincristine solution, Carboplatin solution, Cyclophosphamide solution, Thiotepa solution	• Open-label, non-randomized parallel assignment • Involved 185 participants	• The study focused on treating unilateral RB post-enucleation in accordance with the International RB Staging Working Group.	Phase 2	Ongoing	NCT02870907 [186]
Vincristine solution, Topotecan solution, Filgrastim, Carboplatin solution, Etoposide solution, Cyclophosphamide, Doxorubicin solution	• Non-randomized, parallel assignment • Included 200 participants	• The primary objective was to evaluate the response after systemic topotecan and subconjunctival administration of carboplatin in individuals affected bilaterally. Also, the affected participants had advanced intraocular advanced RB in at least one eye. • The second objective was examining the intraocularly affected tissue, which was undertaken for enucleation. This was done to describe the biallelic inactivation.	Phase 2	Ongoing	NCT01783535 [187]
Topotecan and Melphalan solution	Open-label, randomized, parallel assignment	• Intrathecal administration of melphalan and melphalan + topotecan was evaluated in retinal and diffuse subretinal relapses. • The proposed relapse study period was 10 years to monitor metastasis and secondary malignancies.	Phase 2	Ongoing	NCT04455139 [188]
Topotecan solution and Melphalan solution	• Non- randomized, parallel assignment • Enrolled 50 participants	• The effects of intraocularly administered melphalan and topotecan were studied, and adverse events were also observed during and after the study.	Phase 3	Ongoing	NCT04799002 [189]
Topotecan solution	• Single group assignment • Involved 36 participants	• Selective Intra-ophthalmic artery delivery of topotecan was given to assess the pathway function, pharmacology, and resultant outcomes.	Early phase 1	Terminated	NCT01466855 [190]
Melphalan solution	• Open-label, single-group assignment Involved 10 candidates	• The trial analyzed outcomes after the intravitreal injection of melphalan. The response was found to be transient, with complications persisting over time.	Not applicable	Terminated	NCT01558960 [191]
Melphalan solution, Carboplatin solution, Etoposide solution, Vincristine solution	• Open-label, non-randomized, parallel assignment • Enrolled 6 participants	• The appropriate drug combination was the target of the study after evaluating the safety profile of combined therapy and melphalan therapy at different time intervals. Participants with advanced-stage intraocular RB were selected.	Phase 1	Terminated	NCT02116959 [192]
Melphalan hydrochloride solution	• Single-group assignment • Included 10 participants	• Participants with unilateral or bilateral intraocular RB received intraarterial melphalan hydrochloride intending to limit the aftereffects of systemic chemotherapy and external beam radiation.	Phase 2	Terminated	NCT01293539 [193]
Melphalan solution	• Open-label, single-group assignment • Enrolled 7 participants	• For the administration of melphalan in advanced intraocular RB, the ophthalmic artery was used as the route of administration.	Phase 2	Terminated	NCT01393769 [194]
Carboplatin solution, Topotecan hydrochloride solution, Vincristine solution, Filgrastim solution	• Open-label, single-group assignment	• Topotecan hydrochloride, vincristine, and filgrastim were administered intravenously, along with subtenon administration of carboplatin in cases with a history of bilateral RB at risk of recurrence	Not applicable	Withdrawn	NCT00980551 [195]

## 10 Patents

Innovative cancer treatment methods have been developed worldwide. Table 4 describes various patents and their key findings.

**Table 4:** Various patents approved for the treatment of retinoblastoma (RB).

S. No.	Belonging Country	Type of Formulation	Drug Incorporated	Key Outcomes	References
1.	European Patent	Lyophilized powder	Melphalan	• Upon lyophilizing the melphalan flufenamide solution with sucrose, a massive increase in the solubility was observed. • Thus, making it favourable for physiological fluids. • The amount of sucrose added contributed positively to the dissolution of the preparation.	[197]
2.	Chinese Patent	Lipidosome Injection	Etoposide	• Stability and solubility were found to be better. • The drug retention in the blood was prolonged, contributing to the therapeutic efficacy of the drug.	[198]
3.	Chinese Patent	Nanostructured lipid carrier	Doxorubicin	• Folic acid conjugation helped better target the doxorubicin hydrochloride and gambogic acid-loaded preparation. • The tumour inhibitory effect increased noticeably, adding to the cytotoxicity of the lipid carrier preparation.	[201]
4.	Chinese Patent	Liposomes	Carboplatin	• Noticeable leukocytosis is achieved by cutting down the adverse effects like leukopenia. • The formulation triggered the stimulation of colony growth 12 fold. • Encapsulated carboplatin stimulated hematopoiesis and thus can be used in combination with chemotherapy.	[204]
5.	US Patent	Targeted Liposomes	Melphalan	• Refined anti-cancer efficacy was observed when compared to the native drug solution. • The IC50 value of the liposomes significantly went two folds down.	[196]
6.	US Patent	Liposomal Nanoparticles	Doxorubicin	• The cytotoxicity of the dual drug-loaded nanoparticles was superior to the single drug-loaded nanoparticles. The encapsulation resulted in minimized systemic toxicity levels and was able to inhibit the growth of the tumour significantly. • The formulation delivered drugs at the target site, and a synergistic effect was obtained.	[199]
7.	US Patent	Gold targeted nanoconjugates	Doxorubicin	• Drug-loaded Au-targeted nanoparticles were potently cytotoxic compared to the free drug, and the levels hiked 3000 folds. • Two targeting agents, thioctic acid terminated peptide and bombesin peptide, were used in comparative and combination analysis preparations.	[200]
8.	French Patent	Nanoplexes	Doxorubicin	• Primary and secondary targeting agents hiked up the blood-drug levels of Doxorubicin than the native drug. • DNA as a conjugating agent aided in massive tumour destruction, improving the efficacy manifold.	[203]

Patents on liposomes, targeted nanoconjugates of gold, nanostructured lipid carriers, nanoplexes, and liposomal nanoparticles are outlined below.

### 10.1 Melphalan targeted liposomes

Chang et al. [196] Click or tap here to enter text. used the single-chain anti-transferrin receptor Fv as a ligand to form a complex with the cationic melphalan liposome. *In vitro* studies demonstrated that the anticancer efficacy of melphalan encapsulated in liposomes was higher than that of non-encapsulated melphalan. The IC_50_ value of the conjugated nanocomplex was found to be 2-fold lower than that of native melphalan. It was also found that the IC_50_ value of the nanocomplex was 30 % lower than that of other nanocomplexes prepared with a different molar ratio. These findings suggest that the use of targeted liposomes could potentially improve the effectiveness of melphalan in cancer treatment.

### 10.2 Melphalan-flufenamide lyophilized preparation

Spira et al. [197] Click or tap here to enter text. patented the lyophilized preparation of melphalan-flufenamide that displayed improved solubility in physiological fluids, resulting in a dissolution peak. The dissolution was significantly improved, which could be beneficial in hindering the degradation of melphalan-flufenamide.

### 10.3 Etoposide liposome injection

Yaping et al. [198] Click or tap here to enter text. have developed an etoposide liposome injection procedure. *In vitro* studies demonstrated that the formulation exhibits slow and sustained release behaviour compared to the release behaviour of the crude drug.

### 10.4 Dual drug-loaded liposomal nanoparticles

Bilgicer et al. [199] developed a novel system of doxorubicin and carfilzomib-loaded liposomal nanoparticles. Their study of drug release from the nanoparticles clearly demonstrated a gradual and sustained release of both drugs for up to 72 h. Notably, doxorubicin was released more rapidly than carfilzomib. The cytotoxicity of the nanoparticles was higher than native doxorubicin + carfilzomib and nanoparticles of doxorubicin + nanoparticles of carfilzomib when used separately. The nanoparticles loaded with both drugs showed increased efficacy due to their synergistic effect. In addition, the nano-formulation inhibited tumour growth more than the single nanoparticles.

### 10.5 Targeted doxorubicin-gold nanoconjugates

Kannan et al. [200] filed a patent for doxorubicin conjugated with a targeted gold nano preparation for treating tumours. In this approach, a peptide with a thioctic acid termination was used as a targeting agent, and in some cases, a bombesin peptide was also employed to target the gold nanoparticles better. The nanoparticles thus prepared had a more significant cytotoxic effect than free doxorubicin.

### 10.6 Folic acid-targeted doxorubicin nanostructured lipid carrier

Zhidong et al. [201] Click or tap here to enter text.disclosed a novel invention of a folic acid-targeted nanostructured lipid carrier preparation of doxorubicin hydrochloride and gambogic acid. The formulation exhibited a significant increase in tumour inhibitory effect and enhanced cytotoxicity, attributed to the two drugs' synergistic effect. The IC_50_ value of the nanoformulation was found to be comparatively lower, and the tumouricidal effect of the formulation was also improved upon modification with folic acid.

### 10.7 Doxorubicin-polybutylcyanoacrylate nanoparticles

Yangde filed a patent application for the preparation method of doxorubicin-polybutylcyanoacrylate nanoparticles [202]. Click or tap here to enter text. Nanoparticles have the property of carrying multiple drugs, which would improve the efficiency of targeting and reduce the toxicity of antineoplastic drugs in the body. In addition, the curative effect of the encapsulated drugs is increased, the dosage of drugs is reduced, and the intracellular concentration is also improved.

### 10.8 Doxorubicin nanoplexes

Mixson et al. [203]Click or tap here to enter text. presented an invention regarding targeted doxorubicin-loaded nanoplexes. Doxorubicin is associated with some serious cardiac side effects. These nanoplexes consist of a DNA-conjugated chemotherapeutic agent and a primary and a secondary agent that targets the tumour, resulting in a 5.5-fold increase in the therapeutic concentration of doxorubicin compared to native doxorubicin. The anti-tumour efficacy was significantly improved, as evidenced by the reduced tumour size and higher apoptosis rate. The release of the drug from the nanoplexes critically depends on the degradation of the plasmid DNA, providing a controlled drug release mechanism. Moreover, when the doses of the targeted agents were increased, no toxicity was observed during the *in vivo* activity, highlighting the safety and effectiveness of this approach. Thus, doxorubicin conjugated with DNA exerts a synergistic effect on tumour destruction.

### 10.9 Carboplatin liposomes

Chun et al. [204] Click or tap here to enter text. have developed liposomes loaded with carboplatin. The efficiency of the drug has been shown to be improved after encapsulation of carboplatin in the lipid core. The drug can be effectively incorporated by cooling it to a freezing temperature. Thus, the technique used in this invention can be extended on a large scale.

## 11 Conclusion and future prospectives

Treating RB has always been a Herculean task, with new treatment options emerging every day. The genetic background has been instrumental in developing an effective therapeutic regimen. Understanding various dysregulated pathways responsible for the initiation and progression of RB, small molecule inhibitors which could target different molecular pathways or the cell cycle checkpoints would help to treat RB at the root level and would obstruct the derailing of the normal functioning of the pathways. Following this, clinical research on gene therapy for treating RB would open new horizons in the therapy of RB. Gene editing tools like CRISPR-Cas9 have been studied so far only on the Xenopus tropicalis model and human stem cell lines. This tool seems promising and needs to be explored in depth to move towards effective and harmless treatment for RB. Another effective approach would be using RNAi (interferences) therapeutics for silencing the defective or mutated genes in RB patients. RB Staging systems have also played an important role in its diagnosis. Over the years, these systems have evolved with advances in treatment modalities. The treatment regimen must be adapted according to the size of the tumour and type of RB in accordance with the classification schemes followed worldwide. Conventionally used chemotherapy options such as systemic chemotherapy and focal therapy have certain drawbacks that negatively affect the life of the child. To improve the overall cure rate and mitigate the associated drawbacks, there is a dire need to develop novel drug delivery systems, namely ligand-conjugated nanoparticles, polymeric nanoparticles, nanogels, and dendrimeric nanoparticles. Nanoscale drug delivery systems would deliver the desired effect to the target site, contribute to the therapeutic efficacy of the drug, and thereby minimize adverse effects. Treatment of RB has changed dramatically with the introduction of new methods of administering chemotherapy directly into the eye, such as intra-arterial, intravitreal, and, more recently, intracameral injections, to reduce the frequency of systemic chemotherapy. Additionally, the treatment of RB has advanced significantly with the discovery of gene therapy and immunotherapy for treating this deadly tumour. Both therapies could eradicate the tumour at its root, which in turn would help preserve the vision of children worldwide. The future of cancer therapies lies in the fusion of conventional therapies with nanomedicine, advanced genetic profiling of neonates, nano-immunotherapy and combination therapies. In addition to gene therapy, this review highlights several next-generation strategies, such as new drug molecules, previously unknown potential targets, and novel galenic formulations of existing drugs emerging as potential treatments for RB. Clinical trials of various other drug combinations, routes of administration, and novel formulations are ongoing and suggest improved outcomes. Moreover, these pending clinical trials and patents suggest that dramatic innovations are underway to treat this rare disease. The overexpressed tumour targets discussed in this review, CD24, the HMGA protein, and the nucleolin protein, would disclose new horizons for further research related to this disease.

## Funding

No funding was received for this work.

## CRediT authorship contribution statement

**Ashutosh Pareek:** Writing – review & editing, Writing – original draft, Resources, Project administration, Methodology, Formal analysis, Conceptualization. **Deepanjali Kumar:** Writing – original draft, Software, Methodology. **Aaushi Pareek:** Writing – original draft, Validation, Software. **Madan Mohan Gupta:** Writing – review & editing, Software. **Philippe Jeandet:** Writing – review & editing, Software, Data curation. **Yashumati Ratan:** Writing – review & editing, Software, Formal analysis. **Vivek Jain:** Writing – review & editing, Validation, Formal analysis. **Mohammad Amjad Kamal:** Writing – review & editing, Validation. **Muhammad Saboor:** Writing – review & editing, Validation. **Ghulam Md Ashraf:** Writing – review & editing. **Anil Chuturgoon:** Writing – review & editing, Validation, Methodology.

## Declaration of competing interest

The authors declare that they have no known competing financial interests or personal relationships that could have appeared to influence the work reported in this paper.
